# Multilevel factors influencing parental food decision-making across childhood stages in China: a qualitative study using a socioecological framework

**DOI:** 10.1136/bmjph-2025-004433

**Published:** 2026-09-12

**Authors:** Pui Yee Tan, Peter Yiga, Yiwen Bao, Yun Yun Gong, Rui Ma, Hannah Ensaff, Min Yang, Jili Chen, Jian Huang, Zhenyu Yang, Ou Wang, Junsheng Huo, Gangqiang Ding, Zhaolong Gong, Xiaodong Lin

**Affiliations:** 1 School of Food Science and Nutrition, Faculty of Environment, University of Leeds, Leeds, UK; 2 School of Public Health, and Center of Clinical Big Data and Analytics of The Second Affiliated Hospital, Zhejiang University School of Medicine, Hangzhou, Zhejiang, China; 3 National Institute for Nutrition and Health, Chinese Center for Disease Control and Prevention, Beijing, China; 4 Global Sustainable Development, University of Warwick, Coventry, UK

**Keywords:** Public Health, Qualitative Research, Obesity

## Abstract

**Introduction:**

China faces a double burden of childhood malnutrition. Parents are key dietary gatekeepers, yet limited research has examined how food decision-making evolves across child developmental stages. This study explored multilevel, dynamic determinants of parental food choices across infancy, preschool and primary school age in Zhejiang, China.

**Methods:**

Using visual ethnography, 29 caregivers completed 7-day photographic food diaries followed by in-depth interviews. Data were thematically analysed using an inductive–deductive approach within a socioecological framework. A causal loop diagram was developed to examine cross-level interactions.

**Results:**

Feeding strategies shifted from strong parental control in infancy to increasing child autonomy during preschool years and to time-pressured, convenience-oriented routines in primary school age, often with greater reliance on energy-dense, processed or takeaway foods. Picky-eating strategies evolved from direct food and texture modifications in infancy to gradual exposure and experiential methods in older children. Across stages, parental decisions were shaped by interacting influences including child health priorities, food safety concerns, time constraints, nutrition knowledge and children’s preferences. Grandparents, cultural food norms, traditional beliefs and digital information environments could either support or undermine healthy feeding. Systems analysis further identified reinforcing pathways, including time pressure, convenience reliance, greater child autonomy, grandparent caregiving (eg, indulgent practices), unclear age-specific guidance and digital marketing or misleading online information, that may sustain less healthy practices. In contrast, balancing pathways, including parental prioritisation of child health, stronger nutrition knowledge and trust in school meals and health professionals, supported healthier behaviours, including dietary diversity, home-cooked meals and appropriate supplement use.

**Conclusions:**

Parental food decision-making is developmental, dynamic and shaped by interacting socioecological influences. System-based, age-specific interventions should provide clear guidance through trusted institutions, engage parents and grandparents, strengthen food safety and healthy food environments, regulate digital marketing of unhealthy foods and improve nutrition transparency to support healthier childhood diets in China.

**WHAT IS ALREADY KNOWN ON THIS TOPIC**

- China’s rapid nutrition transition has led to rising childhood overweight and obesity, alongside persistent undernutrition and micronutrient deficiencies, contributing to a growing double burden of malnutrition. Parents are the primary dietary gatekeepers, shaping children’s diets through food purchasing, preparation and feeding practices. However, existing research in China largely focuses on single age groups or individual feeding behaviours, with limited understanding of how parental food choices evolve across childhood or how broader socioecological factors, including family/household, community/organisational and societal influences, shape these decisions.

**WHAT THIS STUDY ADDS**

- Parental feeding practices are developmental and dynamic, shifting from strict control and separate meals in infancy to shared family meals and increasing child autonomy in preschool, and to time-pressured, convenience-oriented routines in primary school age, often with greater reliance on energy-dense, processed and takeaway foods. Food decisions are shaped by interacting influences across the socioecological spectrum, including individual (health priorities, food safety concerns, time constraints, nutrition knowledge, children’s preferences), household and cultural (grandparental involvement, food traditions, social dining) and societal levels, where strong trust in institutions coexists with confusion driven by digital marketing, misleading online information and limited age-specific guidance, particularly for older children. A systems perspective shows reinforcing pathways (eg, time pressure, convenience reliance, grandparent caregiving, digital marketing or online misleading information) that may sustain less healthy practices, and balancing pathways (eg, parental prioritisation of child health, nutrition knowledge, trusted institutions) that support healthier behaviours, including dietary diversity, home-cooked meals and appropriate supplement use.

**HOW THIS STUDY MIGHT AFFECT RESEARCH, PRACTICE OR POLICY**

- Findings support systems-based, age-specific and culturally tailored interventions to improve childhood diets in China. Clearer dietary and supplement guidance, particularly for school-age children, should be delivered through trusted institutions, including schools and healthcare services, and actively engage both parents and grandparents to align feeding practices with nutritional guidance. Policies should aim at strengthening food safety systems, regulating digital marketing of unhealthy foods targeting children and enhancing nutrition transparency, while adopting a systems-based approach to address the multilevel and interacting drivers shaping children’s diets and health outcomes.

## Introduction

 Childhood nutrition plays a pivotal role in long-term health outcomes, influencing physical growth, cognitive development and the risk of chronic diseases in adulthood.^1,2^ China’s rapid economic development and urbanisation have accelerated dietary transitions and reshaped children’s diets.^3,4^ Traditional plant-based diets have increasingly shifted toward animal-source foods, energy-dense and nutrient-poor processed foods and away-from-home meals, alongside declining use of steaming and boiling in favour of frying.^5^ Coupled with reduced physical activity, these changes have contributed to a double burden of malnutrition (DBM), characterised by the coexistence of undernutrition, including micronutrient deficiencies, alongside rising overweight and obesity.^6^

China has achieved substantial reductions in childhood undernutrition, with stunting in children under 5 declining from 28.1% in 1990 to 11.6% in 2016, but progress has slowed down since 2019.^7,8^ Concurrently, childhood overweight and obesity have doubled from 8.5% to 18.8% during the same period, with the steepest rise among school-aged children (6–11 years), increasing from 6.5% in 1991 to 20.7% by 2015.^7,9,10^ Regional data from Zhejiang further highlight higher rates in urban areas (25.8%) than rural areas (20.5%).^11^ Emerging evidence shows that dietary patterns among Chinese children have become increasingly obesogenic, with growing consumption of sugar-sweetened beverages, snacks, processed meats and fried foods, particularly among school-aged children.^12–14^

Children’s diets are shaped by interconnected socioecological factors, as conceptualised in UNICEF’s *Innocenti* Framework.^15^ Parents play a central role as food purchasers, dietary gatekeepers and behavioural models.^16,17^ However, nutritional needs and feeding dynamics evolve as children grow. Infants (6–23 months) transition to complementary feeding while continuing breastfeeding.^18^ During preschool years (3–5 years), children begin developing autonomous feeding behaviour and setting boundaries on food acceptance, and responsive feeding practices, such as repeated exposure to varied flavours and textures, and avoiding overly restrictive practices, can help establish healthy eating behaviours.^19^ Sensory-based learning and hands-on activities, such as cooking and gardening, can further reduce food neophobia and promote dietary diversity.^20^ By primary school age, while familial influences persist, children’s food choices are increasingly shaped by peers, school environments and broader media and marketing exposure.^21^

Despite this central role, most existing research in China has focused on single age groups or individual feeding behaviours, offering limited insights into how parental food decisions evolve across child developmental stages or how household, cultural and broader societal factors shape these decisions. To address this gap, this study used visual ethnography combining 7-day photographic food diaries and in-depth interviews, to explore multilevel determinants of parental food choices across infancy (6–23 months), preschool age (3–5.9 years) and primary school age (6–11 years) in China, guided by a socioecological framework spanning individual, family/household, community/organisational and societal levels.

## Methods

### Study design and study population

The qualitative study followed the Standards for Reporting Qualitative Research (SRQR) (online supplemental table 1). It was conducted between November and December 2022, in Hangzhou, Zhejiang Province, China, within a broader project addressing childhood DBM using food system-based approaches. A visual ethnography design combining 7-day photographic food diaries and in-depth interviews was used to explore parental feeding practices and food decision-making across childhood stages.

Purposive sampling was used to recruit caregivers of children across three age groups: infants (6–23 months), preschool children (3–5.9 years) and primary school-aged children (6–11 years), through Maternal and Child Health centres, preschools and primary schools, respectively, across urban and semiurban/rural communities in Hangzhou to capture diversity in household contexts and food environments. Invitation letters were distributed through participating sites, and interested caregivers contacted the research team. Where families had multiple children, responses referred to the youngest child.

### Data collection

#### 7-day photographic food diary

Participants were asked to document food-related practices over seven consecutive days using their smartphones. They were instructed to capture (1) all foods and beverages prepared or provided to the child, including main meals, snacks and supplements, (2) food procurement sources and (3) food preparation processes and final dishes. Participants submitted approximately 20–30 photographs per day and received detailed instructions to promote consistency. Participants were asked to cover all relevant domains across the 7 days, although daily distribution of photographs across domains varied according to relevance and routine activities. This diary generated real-time records of feeding practices, reduced recall bias and informed subsequent interviews.

#### In-depth interviews

Following diary completion, semistructured interviews were conducted using participants’ photographs as visual elicitation tools.^22^ A subset of photographs was selected for discussion where they best illustrated key practices, contextual influences or contrasting experiences relevant to participants’ food decision-making. Interviews lasted approximately 30 min and were conducted by four trained researchers with backgrounds in nutrition and social science and experience in qualitative child-feeding research in China. Each session involved one facilitator and one note-taker using a standardised protocol. Neutral probing and non-judgemental language were used to minimise social desirability bias. The interview guide (online supplemental table 2) was informed by study aims and prior literature. Topics included food choices, purchasing practices, meal preparation, feeding strategies, views on specific foods and supplements and perceptions of school meals and wider food environments. Open-ended questions were followed by adaptable probes tailored to participants’ photographs.

### Data analysis

All interviews were audio-recorded and transcribed verbatim in Chinese by two native speakers. Transcripts were cross-checked by the lead researchers against recordings for accuracy. Analysis was conducted in Chinese to preserve linguistic and cultural meaning. Key themes and selected quotations were translated into English by bilingual researchers and reviewed through iterative forward-and-back checking to ensure conceptual equivalence. Data were analysed using Braun and Clarke’s six-step thematic analysis framework, with NVivo V.14 software (QSR International, 2014).^23^ A hybrid inductive-deductive coding approach was applied.^24^ Deductive coding was guided by literature on parental food practices, while inductive coding captured emerging concepts. Codes were subsequently grouped into broader themes and mapped onto an adapted socioecological framework defined by McLeroy *et al*, capturing influences at individual, family/household, community/organisational and societal levels.^25^

To strengthen credibility, methodological triangulation was achieved by combining photographic diaries with interview narratives. Two researchers independently coded transcripts, and two additional researchers reviewed coding, met regularly to compare interpretations, refine the coding framework and resolve discrepancies through consensus. Reflexive discussions were conducted throughout the analysis to consider how researchers’ disciplinary backgrounds and assumptions might shape interpretation. Recruitment and preliminary analysis proceeded iteratively. Sampling was considered sufficient when no new major codes emerged and thematic patterns became recurrent across participants.

To further examine cross-level interactions, themes were synthesised into a causal loop diagram (CLD) illustrating how multilevel determinants interacted to shape parental food decisions. Key variables were identified from the thematic analysis and mapped using Visual Paradigm software. Directional arrows represented hypothesised causal relationships, with positive (+) and negative (−) signs indicating the direction of influence. Feedback loops were then classified as reinforcing (R) or balancing (B) and refined through an iterative team-based process.

## Results

### Participants’ characteristics

30 caregivers were recruited, but one withdrew before interview completion, leaving 29 interviews for analysis. Demographic data were unavailable for one participant, resulting in n=28 for table 1 variables. The mean child’s age was 4±5 years, with 60.7% being males, 57.1% being only children, 67.9% lived in semiurban or rural areas and 71.4% co-resided with grandparents. Most caregivers were females (96.4%), parents (64.3%), working adults (60.7%) and had completed high school or above (89.3%). Nutritional supplement use was common (71.4%).

**Table 1:** Demographic characteristics of the study participants

Demographic variables	Total (N=28)	
	Number	%
Child’s age (years±SD)	4±5	
Age group		
Infants (6–24 months)	9	32.1
Preschool (3–5 years)	9	32.1
Primary school (6–11 years)	10	35.8
Child’s sex		
Male	17	60.7
Female	11	39.3
Region		
Urban	9	32.1
Semiurban or rural	19	67.9
Ethnicity		
Han (majority)	26	92.9
Other minorities	2	7.1
Only child		
Yes	16	57.1
No	12	42.9
Primary caregiver		
Parents	18	64.3
Grandparents	10	35.7
Caregiver’s sex		
Male	1	3.6
Female	27	96.4
Caregiver’s educational level		
Junior high school or below	3	10.7
High school or college graduate	12	42.9
University graduate and above	13	46.4
Primary caregiver’s occupation		
Working	17	60.7
Non-working (retirees, housewives)	11	39.3
Co-living with grandparents		
Yes	20	71.4
No	8	28.6
Supplementation		
Yes	20	71.4
No	8	28.6

### Overview of feeding practices across developmental stages

**Figure 1:**
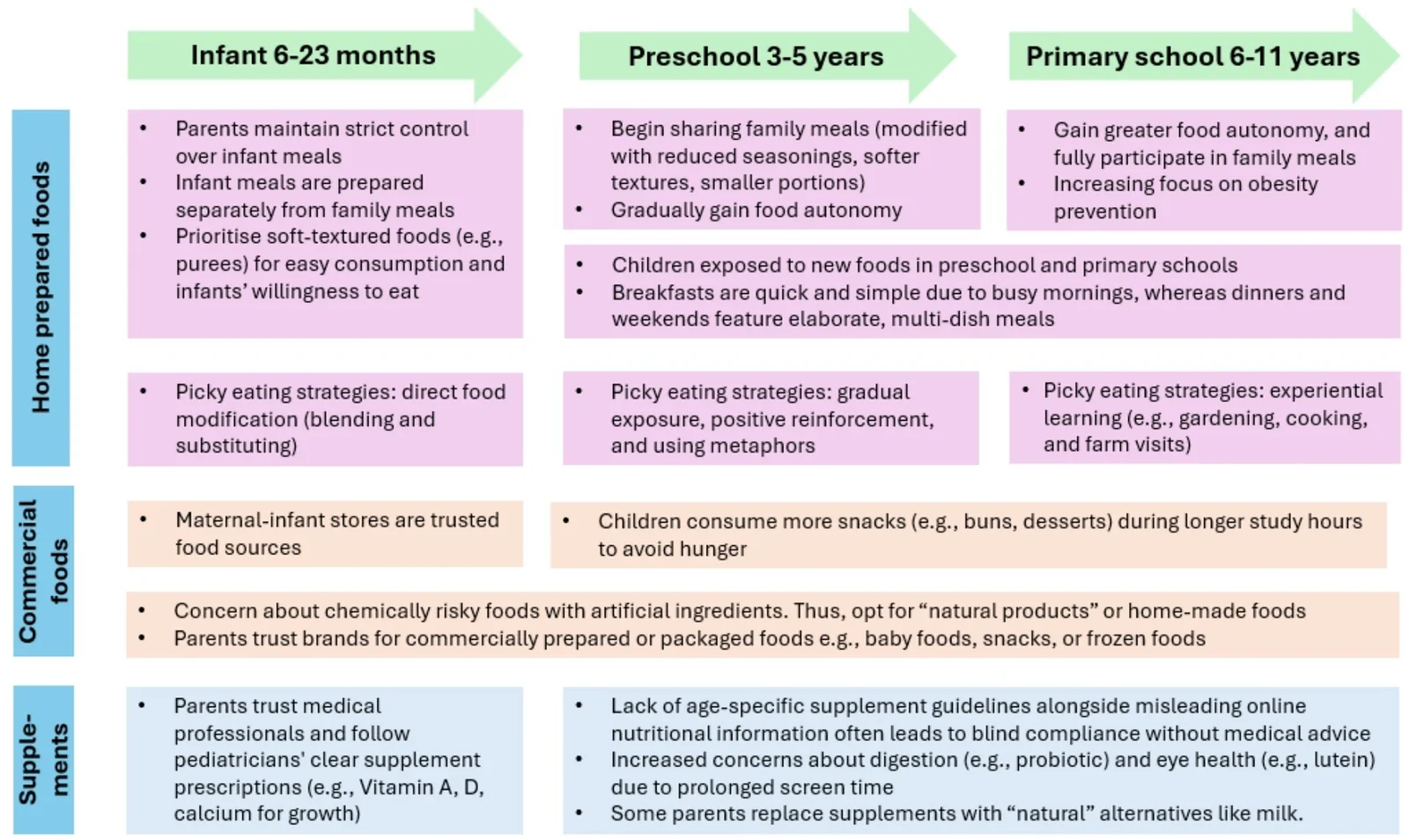
Parental feeding practices and food choices across children’s developmental stages by food type.

Feeding practices varied across childhood stages (figure 1). During infancy (6–23 months), caregivers maintained high control over feeding, preparing separate soft-textured meals (eg, purees, milk powder and infant-specific snacks sourced from trusted stores) and following paediatric supplement advice. In preschool (3–5 years), children gradually gained autonomy while joining family meals, with modifications such as reduced seasoning, softer textures and smaller portions. By primary school age (6–11 years), children had greater autonomy in food choices, however, caregivers increasingly relied on convenience (eg, simplified, quick breakfast) and processed foods (eg, snacks) due to time constraints. Supplement use also became less medically guided and more influenced by online information or personal judgement. Approaches to picky eating also evolved from direct food modifications (eg, blending or substituting foods) in infancy to gradual exposure and storytelling during preschool, and hands-on strategies such as cooking, gardening, in later childhood.

### Multilevel influences on parental food choices

**Figure 2:**
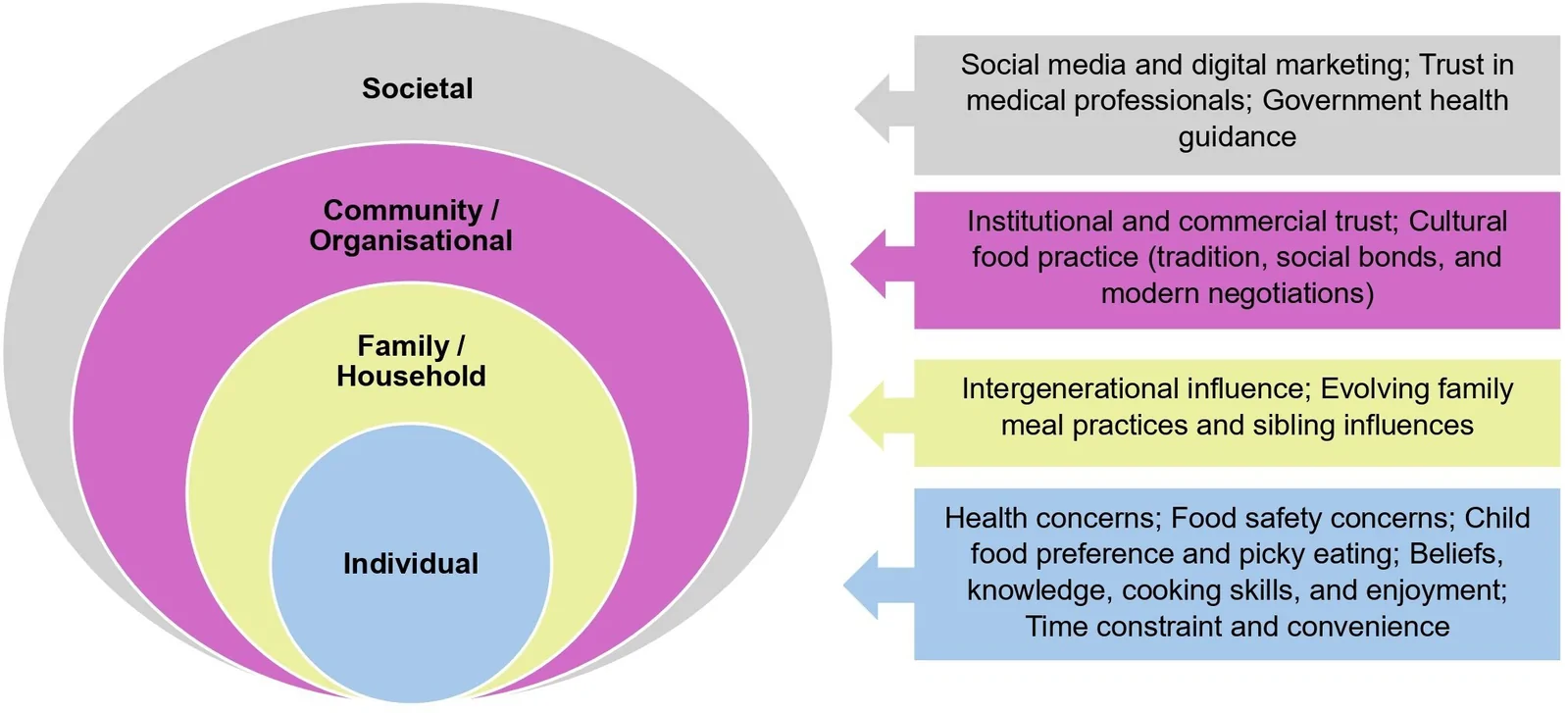
Multilevel determinants influencing parental food decision-making, organised using an adapted socioecological framework.

Findings were organised using a socioecological framework across individual, family/household, community/organisational and broader societal levels (figure 2). Themes are described below alongside participant quotes (eg, Parent of ‘child age group’—sequence number).

#### Individual-level factors

##### Health concerns: growth, obesity and other specific concerns

Across all child age groups, caregivers prioritised child growth, health and balanced diets (eg, preventing stunting, overweight, obesity and illness, or improving eye or digestive health). These priorities shaped efforts to provide vegetables, fruits and protein-rich foods such as beef, poultry and milk, and calcium supplements, where perceived necessary. As one parent explained: “We insist on having vegetables every day, along with meat like beef or pork, because I want him [son] to grow taller.” (Parent of infant 3). By primary school years, parents increasingly expressed concerns about overweight and obesity, leading to restrictive practices such as portion control, scheduled snacking and substituting high-calorie snacks with healthier alternatives (eg, nuts, fruits, milk and yoghurt). Parents also used supplements for child-specific needs, especially in older children, including lutein (for eye health due to prolonged study and screen time), vitamin C (to prevent illness) and probiotics or traditional Chinese remedies (for digestive health).

##### Food safety concerns

Food safety concerns were widespread, particularly regarding perceived links to early puberty and exposure to harmful substances (eg, hormones, pesticides, additives and genetically modified crops). Parents reported avoiding foods perceived to contain growth stimulants (eg, chicken wings, eggs and farmed shrimp). In response, caregivers often prepared homemade meals, selected trusted or certified brands (eg, organic or hormone-free), checked food labels and were willing to pay premium prices for products perceived as safer, especially for infant foods or snacks. Wet markets were preferred for fresh produce due to affordability and convenience, while trusted supermarkets were preferred for meat and packaged goods due to perceived stricter quality and hygiene standards. Some families also relied on home-grown produce from neighbours or rural relatives. Fresh produce purchased online was often viewed with caution, particularly by grandparents, while a few caregivers felt such risks were unavoidable regardless of the source.

##### Child food preferences and picky eating

Children’s taste preferences emerged as a significant factor influencing parental food selection. Picky eating, particularly rejection of vegetables, mushrooms and certain meats, was common. Parents adopted a range of coping strategies that evolved with age. During infancy, parents primarily relied on direct modification strategies, such as substituting disliked foods with alternative vegetables or meat sources, blending them with preferred foods, or adjusting textures and cooking methods. In preschool years, strategies shifted toward parental role modelling, gradual exposure, and positive reinforcement. Many parents also used child-friendly metaphors or storytelling to explain the importance of balanced nutrition. As one parent noted *“*A colourful painting is beautiful, just like your body needs colourful nutrients too.” (Parent of preschool 5). Some parents of older children viewed hands-on experiences, such as gardening, farm visits, or involving them in meal preparation, as effective for reducing food neophobia and increasing food acceptance.

##### Beliefs, knowledge, cooking skills and enjoyment

Parental feeding practices were strongly influenced by core nutritional beliefs, particularly the importance of dietary ‘balance’ between meat and vegetables and dietary variety to provide a range of nutrients. Many parents associated animal-source foods with physical growth and vegetables with micronutrients and fibre. However, translating these beliefs into practice depended significantly on parental knowledge, culinary skills, and confidence. Caregivers with stronger knowledge or cooking enthusiasm described preparing more varied, nutrient-dense meals and reported greater satisfaction when children ate well. Conversely, caregivers with limited skills relied more on traditional, intergenerational knowledge, resulting in simpler, repetitive dishes.

##### Time constraints and convenience

Modern work schedules and school routines significantly influenced meal preparation. On time-scarce weekday mornings (eg, work commitments or school drop-offs), most caregivers relied on quick and simple breakfasts, such as noodles, steamed buns, frozen dumplings, or fried starchy foods from street vendors, particularly in households with school-aged children. Whereas evenings and weekends typically featured more elaborate, multicourse meals with diverse ingredients. Grandparent support helped reduce time pressure, especially for working parents. Without such support, parents reported greater reliance on repetitive dishes, convenience foods, takeaway options, or preprepared foods, especially after long workdays. Families living further from traditional markets also used e-commerce for routine groceries or specialty items.

### Family or household-level factors

#### Intergenerational influence: the role of grandparents

Grandparents played a complex and multifaceted role in shaping children’s diets, especially in multigenerational households. Parents consistently valued grandparents’ culinary expertise, food selection skills, and childcare support. As one participant noted, “Grandma knows how to tell good meat from bad. We, the younger generations, are clueless.” (Parent of primary school-aged children 4). Grandparents also tend to preserve culinary traditions, maintaining cultural recipes and cooking techniques passed down through generations. However, tensions sometimes arose where grandparents preferred richer flavours (higher salt, oil and sugar), larger portions or indulgent feeding practices (eg, providing snacks) often conflicted with modern health guidance. Parents described navigating this tension by maintaining respect for elders and accepting grandparents’ practices, especially when time constraints reduced opportunities to negotiate changes.

#### Evolving family meal practices and sibling influences

Parents described distinct feeding practices by child age. Infants often received separate meals, such as purees, while preschoolers were gradually introduced to family foods with adaptations, such as reduced seasoning, softer textures or smaller portions. By primary school age, they increasingly participated in shared family meals and exercised greater autonomy in meal choices. To accommodate varying tastes, caregivers often ensured each meal included at least one preferred dish for every family member, helping avoid conflict and minimising the need for multiple meal preparations, particularly in larger families. Sibling dynamics further shaped food behaviours. Parents in households with multiple children described that younger siblings tended to model older siblings’ eating habits and snack choices, including willingness to try new foods. Parents also reported adapting feeding approaches for later-born children based on prior experience.

### Community or organisational-level factors

#### Institutional and commercial trust

Caregivers expressed strong trust in two institutional settings: school meals and mother-infant specialty stores (eg, formula milk, baby snacks, supplements). School meals were consistently viewed as safe, diverse and nutritionally balanced due to perceived government oversight, standardised procurement, rotating menus and transparency (eg, teachers sharing daily school meals via group chat). As one parent stated, “I trust that public schools maintain decent food safety standards, because there is strict oversight in place.” (Parent of primary school-aged children 3). Parents added that school settings helped address picky eating, as children were more likely to eat foods provided in structured environments. For infants, mother-and-infant stores were widely trusted because products were perceived as superior or higher quality, while staff recommendations were often viewed as credible guidance.

#### Cultural food practices: tradition, social bonds and modern negotiations

Many caregivers emphasised the deep cultural significance of shared meals and communal dining, which symbolise family unity, particularly dinners and weekend family gatherings. Special occasions, such as birthdays, festivals and housewarmings, typically featured abundant, elaborate meals, which also symbolised prosperity and familial well-being. Beyond the household, gift-giving and neighbourly meal sharing strengthened social connections. However, parents noted tensions when gifted foods were unhealthy, yet they felt compelled to accept them to maintain relationships and social harmony. Regional cuisines shaped everyday food practices, with northern-origin families more likely to consume wheat-based foods (eg, dumplings, noodles, steamed buns), whereas native Zhejiang families more often described rice-based meals with lighter seafood dishes. Some caregivers drew on Traditional Chinese Medicine (TCM) principles when selecting foods, for example, balancing ‘heating’ and ‘cooling’ properties, and prioritised seasonal eating aligned with natural cycles, believing these practices supported better health. As one parent explained, “When eating crab, we add ginger or vinegar to counteract its ‘cool-natured’ properties.” (Parent of primary school-aged children 10).

### Societal-level factors

#### Social media and digital marketing

Most parents identified social media platforms (eg, Rednote, Douyin/TikTok, WeChat) as primary sources for nutritional and health tips, recipes, product recommendations and expert advice. Parents expressed trust in peer reviews and influencer content, especially for infant foods, supplements and commercial products. However, some decisions are often shaped by viral health trends rather than scientific recommendations, including blind compliance or inappropriate extended supplement use beyond clinical guidance. Other parents described more critical engagement with digital content, carefully evaluating online information against their own knowledge and judgement. As one parent explained, “Expert recommendations matter, but I filter them and only adopt advice that aligns with my logic.” (Parent of primary school-aged children 6).

#### Trust in medical professionals

Parents consistently identified doctors and paediatricians, as trusted sources for dietary and supplement advice. This was particularly evident among mothers of infants (6–23 months), nearly all of whom reported adherence to prescribed vitamin A, D or calcium regimes during clinical visits. Parents also described adhering to dietary advice from healthcare providers to support their children’s nutritional needs. However, some parents continued supplement use beyond the recommended duration or medical advice based on personal beliefs or perceived benefits, illustrating how professional advice sometimes merged with parental intuition.

#### Government health guidance

Parents of infants frequently referenced official nutrition guidance from (eg, Maternal and Child Health) and used these resources for daily meal planning, bridging clinical recommendations with home practice. As one participant noted, ‘‘Our local Health Bureau offers a free Maternal and Child Nutrition programme (WeChat) that sends daily meal ideas. I often borrow recipes from it.” (Parent of infant 1).

**Figure 3:**
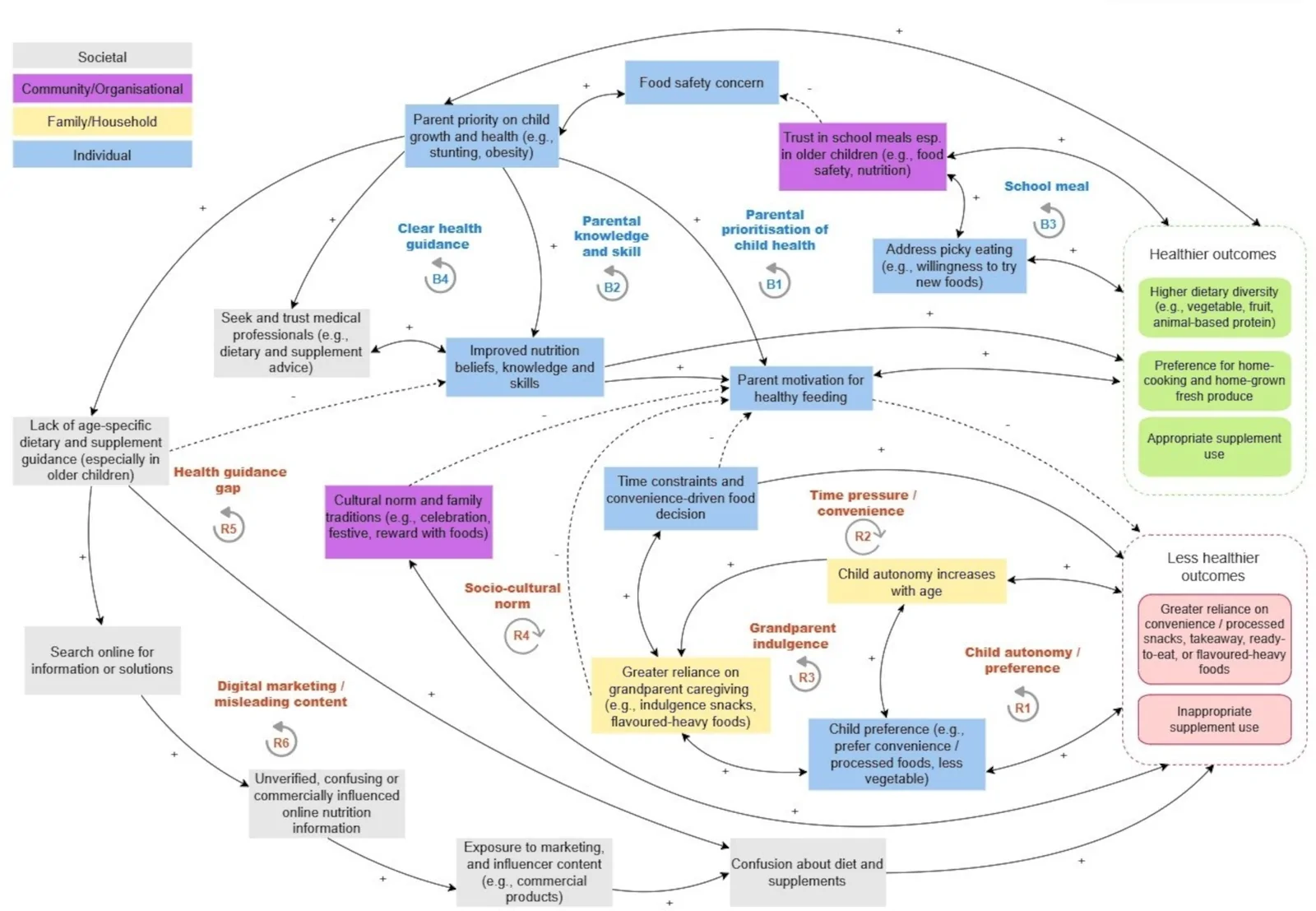
Causal loop diagram illustrating the dynamic pathways influencing parental food decision-making and child dietary outcomes across childhood stages in China.

To further explore how determinants interacted dynamically, a CLD was developed from the qualitative findings (figure 3). The model showed that parental food decision-making was shaped by multiple interconnected drivers, comprising six reinforcing loops (R1–R6) and four balancing loops (B1–B4). Reinforcing loops sustained less healthy trajectories through increasing child autonomy and preferences for convenience or processed foods, parental time pressure, greater reliance on grandparent caregiving associated with more indulgent feeding practices, sociocultural norms, gaps in clear age-specific guidance, and exposure to confusing or commercially influenced online nutrition information and food marketing. In contrast, balancing loops reflected protective mechanisms whereby parental prioritisation of child health, improved nutrition knowledge and feeding skills, trust in school meals and access to trusted medical professionals providing clear age-appropriate dietary and supplement guidance strengthened informed decision-making. Together, these balancing loops may promote healthier dietary outcomes, including greater dietary diversity, preference for home-cooked foods and appropriate supplement use.

## Discussion

This study suggests that parental food decision-making is shaped not only by individual preferences but also by interacting influences across family/household, community/institutional and wider societal and digital environments. Feeding practices also changed across childhood stages, with parental control highest in infancy, increasingly negotiated during preschool years and more constrained by child autonomy, school routines and convenience pressures during primary school age. Consistent with the causal loop analysis, reinforcing pathways (eg, time pressure, convenience-driven food choices, child preferences and autonomy, grandparent caregiving, lack of age-appropriate guidance and exposure to digital marketing on unhealthy foods or confusing online nutrition information) may sustain less healthy feeding practices. In contrast, balancing pathways (eg, parental prioritisation of child health, stronger nutrition knowledge and trust in school meals and access to trusted health professionals) support healthier choices, including greater dietary diversity, home-cooked meals and more appropriate supplement use. Together, these findings highlight that food decision-making is developmental, dynamic and system-driven rather than static across childhood.

At the individual level, caregivers consistently prioritised children’s growth, health and food safety, emphasising varied diets rich in vegetables and protein-rich foods (meat, eggs, milk and dairy) while sourcing foods from trusted outlets. This reflects broader dietary transitions in China, where intake of animal-source foods among children tripled between 1990 and 2019, while urban children consumed more protein and fat than rural peers.^26,27^ Food safety concerns further shaped ‘defensive consumption’ behaviours, including avoidance of perceived risky foods (eg, chicken wings and synthetic additives) and selective sourcing from trusted outlets. Parents commonly purchased fresh produce from wet markets or home gardens while relying on supermarkets for meat and packaged foods, consistent with evidence showing increasing consumer preference for supermarkets, particularly from higher-income families and willingness to pay more for safety-certified foods.^28–30^ However, limited label literacy skills may hinder informed purchasing decisions.^31^

Despite these intentions, healthy feeding was often constrained by limited time, practical demands and uncertainty regarding nutrition information. This may help explain why the greater reliance on convenience foods, takeaway meals and processed snacks became more common as children grew older and family routines became busier. Nearly 80% of children in Chinese mega-cities ate out at least once weekly alongside the rapid expansion of fast-food outlets and the rising obesity rate.^32,33^ Diets high in processed meats and fast foods have also been associated with a doubled obesity risk among Chinese school-aged children.^34^ Picky eating was another widely reported challenge. High prevalence of picky eating was reported among Chinese school-aged children (59.3%) with associated micronutrient deficiencies negatively impacting growth and cognitive development.^35,36^ Parents employed stage-specific coping strategies, including direct food or texture modifications in infancy, gradual exposure and positive reinforcement in preschool years and experiential approaches, such as gardening, cooking or farm visits for older children, which may help reduce food neophobia.^37,38^

Within multigenerational households, grandparents were both protective and constraining influences. Their caregiving support and culinary knowledge reduced pressure for working parents, especially in dual-working households.^39^ However, indulgent feeding practices, richer flavours (higher salt, oil and sugar) and larger portions often conflicted with parental health goals or modern nutritional advice promoting moderation, balanced diets and reduced intake of energy-dense foods. Such conflicts were particularly pronounced in families managing childhood obesity, undermining parental efforts at dietary restraint.^40^ Feeding decisions were therefore often negotiated within family power structures rather than determined by parents alone. As multigenerational living remains common in China, interventions targeting only parents may overlook key household decision-makers.^41,42^ Furthermore, China’s shift to a multichild policy may also introduce new dynamics, with younger siblings often adopting older siblings’ eating or snack habits and parents described adjusting their feeding strategies with subsequent children through experiential learning based on prior experience.^43–45^ Meanwhile, persistent gender norms continue placing the burden of food preparation on mothers and grandmothers despite increasing women’s workforce participation, reinforcing dual caregiving and labour demands.^46,47^

Parents also appeared to navigate tensions between traditional food knowledge and modern nutrition advice obtained from health professionals, official guidance or digital sources. While many caregivers trusted paediatricians and evidence-based recommendations, cultural food traditions retained deep symbolic significance, particularly during festivals, social gatherings and banquets, where elaborate dishes signified prosperity and family unity but often conflicted with health-focused nutrition guidance.^48^ Regional dietary diversity, including contrasts between northern and southern cuisines, also shaped practices.^49^ TCM principles continued to influence food choices, with parents carefully balancing ‘cold’ foods (believed to calm blood and clear toxins) with ‘hot’ foods (thought to improve circulation) when selecting foods for children.^50,51^ These findings suggest that parental decisions are influenced not simply by evidence-based recommendations versus conflicting or commercially influenced information but by multiple systems of trust, culture and perceived credibility.

Several institutional settings emerged as trusted leverage points. Parents commonly expressed confidence in doctors or paediatricians, Maternal and Child Health services, school meals and mother–infant stores. During infancy, trust in paediatricians and maternal–child services supported uptake of recommended vitamin A (1500–2000 IU daily), vitamin D (400–800 IU daily) and calcium supplementation, as outlined in the 2024 Expert Consensus, while mother–infant stores were often viewed as reliable sources of infant products.^52^ For older children, school meals were widely perceived as safe and nutritionally balanced due to strict government oversight, standardised procurement channels and transparent practices (eg, shared daily meal photos). This contrasts with Western evidence, where parents often perceive school meals as poor quality, reducing participation rates.^53,54^

Digital platforms increasingly shape parental food decisions through recipes, marketing and promotion, influencer advice and peer recommendations. While some parents critically evaluated online content, others reported reliance on viral health trends or prolonged supplement use beyond clinical advice. This may reflect a digital information environment where commercial incentives, algorithmic amplification and weak regulation blur boundaries between education, advertising and commercially influenced or unverified nutrition information. In particular, the lack of clear, age-specific dietary and supplement guidance may increase caregivers’ reliance on digital sources, thereby increasing exposure to misleading or commercially-driven content. Previous studies found that influencers often prioritised product promotion over evidence-based recommendations, and 37.5% of Chinese parents report difficulty evaluating dietary supplements, with 26% purchasing from unverified online sources.^55,56^ Although regulatory bodies have strengthened oversight of online advertising for health foods and formulas, enforcement remains challenging due to resource constraints (human, technological and financial) and low consumer awareness.^57,58^ Child-targeted digital food marketing remains insufficiently regulated, complicating parents’ ability to distinguish credible advice from commercial promotions.^59^

### Implications for interventions

These findings highlight the need for integrated, developmentally tailored and culturally appropriate strategies aligned with China’s Healthy China 2030 and National Nutrition Plan, which prioritise healthier diets, obesity prevention and improved population nutrition across the life course. Clear, age-specific dietary and supplement guidance should be delivered through caregivers’ trusted channels. During infancy, interventions should strengthen complementary feeding and evidence-based supplement advice through maternal and child healthcare services. During preschool years, programmes could support picky eating management, responsive feeding and healthy family mealtime practices. For school-aged children, strategies should focus on healthier snack environments, digital nutrition literacy and autonomy-supportive approaches that help children make healthier choices.

Interventions should also actively engage grandparents and other caregivers, recognising their influence on household feeding decisions, while addressing gendered caregiving burdens. Schools and primary healthcare settings could provide practical workshops for meal planning, cooking skills, healthy lunchboxes, parent engagement and experiential learning, such as school gardens or cooking classes. At the policy level, priorities include protecting neighbourhood access to affordable fresh produce, strengthening food safety governance, tightening regulation for digital food and beverage marketing, especially targeting children and adolescents, and enhancing online nutrition transparency. Public health authorities could also leverage verified digital platforms, such as WeChat, to provide clear, age-appropriate nutrition and supplement guidance for caregivers and support informed food choices.

### Strengths and limitations

This study has several notable strengths. The use of visual ethnography, combining photo diaries and interviews, generated rich, contextualised data on feeding practices while minimising recall bias. Methodological rigour was strengthened through triangulation of visual and verbal data, multiple coders and analysis of Chinese transcripts to preserve cultural and linguistic nuances. Inclusion of three age groups enabled analysis of age-specific strategies, which are often overlooked in single-age studies. Combining socioecological and systems perspectives strengthened the interpretation of multilevel influences and their interactions.

Several limitations should be considered. Participants were recruited from Zhejiang Province and were relatively highly educated, predominantly female and commonly from multigenerational households, which may limit transferability to less advantaged populations or other regions of China. Fathers, non-working caregivers and left-behind children were also underrepresented. Purposive sampling and voluntary participation may have also attracted caregivers with greater interest or engagement in child health or nutrition. As with all interview-based studies, responses may have been influenced by social desirability bias, with participants potentially presenting feeding practices more favourably. Although visual methods effectively captured behaviours, they provided only a seasonal snapshot of food choices and may not fully reveal implicit decision-making processes requiring longer-term observation. Finally, the cross-sectional design limits causal inferences about how feeding strategies evolve within families over time.

## Conclusions

This study shows that parental food decision-making in China is developmental, dynamic and shaped by interacting influences across family, community, institutional and digital environments. As children grow, feeding often shifts from strong parental control in infancy to increasing child autonomy in later childhood, when time pressure and convenience demands may make healthy choices harder to sustain. In contrast, stronger nutrition knowledge and trusted support from schools and health professionals may help promote more diverse diets, home-cooked meals and appropriate supplement use. Grandparents, cultural food norms and digital information environments were key influences that could either support or undermine healthy feeding practices. These findings suggest that improving childhood diets requires systems-based, age-specific strategies that go beyond individual behaviour change. Policies and interventions should provide clear age-specific guidance through trusted institutional channels, engage both parents and grandparents, strengthen food safety regulation and healthier school and community food environments, regulate digital marketing of unhealthy foods and improve nutrition transparency to support healthier dietary trajectories across childhood in China.

## Supplementary material

10.1136/bmjph-2025-004433 online supplemental file 1

## Data Availability

Data are available on reasonable request.
