# Supplementary material for: Multilevel factors influencing parental food decision-making across childhood stages in China: a qualitative study using a socioecological framework
Source: BMJ Public Health. 2026 Sep 12;4(3):e004433. doi: 10.1136/bmjph-2025-004433 (PMC13583821; doi:10.1136/bmjph-2025-004433)
Supplement: Supplementary file 1 [file bmjph-4-3-s001.docx]

**Multi-level factors influencing parental food decision-making across childhood stages in China: A qualitative study using a socio-ecological framework**

*Pui Yee Tan^1^, Peter Yiga^1^, Yiwen Bao^1^, Yun Yun Gong^1^, Rui Ma^1^, Hannah Ensaff^1^, Min Yang**^2^, Jili Chen^2^, Jian Huang^3^, Zhenyu Yang^3^, Ou Wang^3^, Junsheng Huo^3^, Gangqiang Ding^3^, Zhaolong Gong^3^*, *Xiaodong Lin^4^*

**Supplementary Table 1: Standards for reporting qualitative research (SRQR) checklist**

| No. | Topic | Items | Page no. |
| --- | --- | --- | --- |
| **Title and Abstract** | | | |
| S1 | Title | Concise description of the nature and topic of the study Identifying the study as qualitative or indicating the approach (e.g., ethnography, grounded theory) or data collection methods (e.g., interview, focus group) is recommended | 1 |
| S2 | Abstract | Summary of key elements of the study using the abstract format of the intended publication; typically includes background, purpose, methods, results, and conclusions | 1-2 |
| **Introduction** | | | |
| S3 | Problem formulation | Description and significance of the problem/phenomenon studied; review of relevant theory and empirical work; problem statement | 3-4 |
| S4 | Purpose or research question | Purpose of the study and specific objectives or questions | 4 |
| **Methods** | | | |
| S5 | Qualitative approach and research paradigm | Qualitative approach (e.g., ethnography, grounded theory, case study, phenomenology, narrative research) and guiding theory if appropriate; identifying the research paradigm (e.g., postpositivist, constructivist/ interpretivist) is also recommended; rationale | 4-6 |
| S6 | Researcher characteristics and reflexivity | Researchers’ characteristics that may influence the research, including personal attributes, qualifications/experience, relationship with participants, assumptions, and/or presuppositions; potential or actual interaction between researchers’ characteristics and the research questions, approach, methods, results, and/or transferability | 6 |
| S7 | Context | Setting/site and salient contextual factors; rationale | 4-5 |
| S8 | Sampling strategy | How and why research participants, documents, or events were selected; criteria for deciding when no further sampling was necessary (e.g., sampling saturation); rationale | 5-6 |
| S9 | Ethical issues pertaining to human subjects | Documentation of approval by an appropriate ethics review board and participant consent, or explanation for lack thereof; other confidentiality and data security issues | 5 |
| S10 | Data collection method | Types of data collected; details of data collection procedures including (as appropriate) start and stop dates of data collection and analysis, iterative process, triangulation of sources/methods, and modification of procedures in response to evolving study findings; rationale | 4-6 |
| S11 | Data collection instruments and technologies | Description of instruments (e.g., interview guides, questionnaires) and devices (e.g., audio recorders) used for data collection; if/how the instrument(s) changed over the course of the study | 5-6 |
| S12 | Units of study | Number and relevant characteristics of participants, documents, or events included in the study; level of participation (could be reported in results) | 5 |
| S13 | Data processing | Methods for processing data prior to and during analysis, including transcription, data entry, data management and security, verification of data integrity, data coding, and anonymization/de-identification of excerpts | 6-7 |
| S14 | Data analysis | Process by which inferences, themes, etc., were identified and developed, including the researchers involved in data analysis; usually references a specific paradigm or approach; rationale | 6-7 |
| S15 | Techniques to enhance trustworthiness | Techniques to enhance trustworthiness and credibility of data analysis (e.g., member checking, audit trail, triangulation); rationale | 6 |
| **Results/findings** | | | |
| S16 | Synthesis and interpretation | Main findings (e.g., interpretations, inferences, and themes); might include development of a theory or model, or integration with prior research or theory | 7-13; Table 1; Figures 1-3 |
| S17 | Links to empirical data | Evidence (e.g., quotes, field notes, text excerpts, photographs) to substantiate analytic findings | 8-13 |
| **Discussion** | | | |
| S18 | Integration with prior work, implications, transferability, and contribution(s) to the field | Short summary of main findings; explanation of how findings and conclusions connect to, support, elaborate on, or challenge conclusions of earlier scholarship; discussion of scope of application/generalizability; identification of unique contribution(s) to scholarship in a discipline or field | 13-17 |
| S19 | Limitations | Trustworthiness and limitations of findings | 17-18 |
| **Others** | | | |
| S20 | Conflict of interest | Potential sources of influence or perceived influence on study conduct and conclusions; how these were managed | 19 |
| S21 | Funding | Sources of funding and other support; role of funders in data collection, interpretation, and reporting | 19 |

**Supplementary Table 2: Qualitative interview topic guide (questions and prompts)**

1. **Factors affecting parents’ decisions on foods prepared for their children**

Q1. Can you describe your feelings about preparing these foods for your child during the week?

- Why do you feel this way?

Q2. Can you share some examples of meals or foods you have prepared for your child? (Pick some pictures from photo diary)

- Breakfast, lunch, dinner, and snacks (weekdays vs. weekend)
- What ingredients did you use?
- How would you describe the taste, texture and your child’s reaction?

Q3. What are the most important factors you consider when preparing food for your child? (e.g., cost, seasonality, tradition, nutrition, taste, child’s preference, expert’s advice etc.)

- Why do these factors matter to you?
- How do they influence your food choices?

Q4. Do you cook these meals yourself or buy them?

- If you didn’t cook it yourself: why not, and who?
- If bought: Where from, and why do you choose this place?

Q5. Do you have any concerns or worries about the foods that your child consumes?

- What specific worries do you have when selecting foods? Please give an example.
- How do you address these concerns? Please give an example.

2. **Factors affecting parental food sourcing practices**

Q6. When deciding where to shop for your child’s food, what do you prioritise most? (e.g., proximity, freshness, brand trust etc.)

- Are there differences in where you buy fresh produce vs. packaged foods?
- Why are these aspects important to you?

3. **Parental views on supplementation or other specific foods**

Q7. Do you introduce your child any nutritional supplementary or special foods (e.g., vitamins, fortified foods)?

- Why or why not?
- How do you decide which ones to give?
- Does your child like them? What do you do if they refuse? Please give an example.

4. **Parental views on school meals**

Q8. What are your thoughts on the meals provided at your child’s school?

- Why do you feel this way?

Q9. Do you prepare additional food when they come home?

- Why?

5. **Learning sources for child nutrition**

Q10. Did someone teach you how to select or prepare these foods? Where did you learn it from?

- Any other sources you rely on (e.g., internet, doctors, friends)?
- How do these sources affect your food choices?

6. **Parental perception of their cooking**

Q11. Which dish/recipe do you think you make best for your child?

- Why do you think that?

Q12. On a scale of 1-10, how would you rate the meals or foods you prepared for your children?

- What influences this score?

***You can continue to ask the question from 2-12*
